# Pulmonary Hypertension due to a Pulmonary Artery Leiomyosarcoma: A Case Report

**DOI:** 10.1155/2013/160619

**Published:** 2013-03-31

**Authors:** Seyyed Hassan Adeli, Bardia Nemati, Mahboubeh Jandaghi, Mohammad Mahdi Riahi, Fatemeh Hosseinzadeh, Fatemeh Salarvand

**Affiliations:** ^1^Clinical Research Development Center, Qom University of Medical Sciences, Qom 3719764799, Iran; ^2^Qom University of Medical Sciences, Qom 3719764799, Iran

## Abstract

*Background*. Primary pulmonary artery sarcomas are very rare and their histologic type, called leiomyosarcoma, is even rarer. *Case Report*. A 64-year-old woman presented with progressive weakness, fatigue, malaise, and dyspnea, and a marked elevation of pulmonary artery pressure was admitted. She was initially diagnosed with chronic pulmonary thromboembolism and chest computed tomography (CT) scan revealed that lobulated heterogeneous left hilar mass extended to precarinal and subcarinal space. MRI demonstrated a polypoid lesion at trunk with extension to left main pulmonary artery and its first branch. She was operated, a yellowish-shiny solid mass in pulmonary trunk was seen intraoperatively, and pulmonary endarterectomy was performed. Her tumor was pathologically diagnosed as pulmonary artery leiomyosarcoma. She died 3 months later after one chemotherapy course. *Conclusion*. Initially, the patient underwent surgery due to pulmonary embolism but, during the operation, the observed mass increased the probability of pulmonary artery neoplasm. Clinicians must consider pulmonary artery sarcoma when making the differential diagnosis for patients with pulmonary arteries masses. In addition to clinical prediction scores and CT and MRI findings to identify the patients with pulmonary artery sarcoma, PET scanning is the diagnosis of choice in differentiating embolism and neoplasm and is strongly recommended in these patients.

## 1. Introduction 

Primary pulmonary artery sarcomas are very rare and their histologic type called leiomyosarcoma is even rarer [1]. The incidence of primary pulmonary artery tumors is 0.001–0.03%, and they are nearly always highly malignant and typically obtain their origin from the intima [2]. The underlying pathophysiology of these tumors of the pulmonary arteries is still unclear [2, 3].

These tumors are frequently misdiagnosed as pulmonary thromboembolism in clinical settings. Many patients receive anticoagulant therapy without response, and many are diagnosed postmortem only [4]. Most of the tumors reported in the literature have involved the right ventricular outflow tract and the main pulmonary trunk, often extending into the main pulmonary artery branches [5].

## 2. Case Report 

**Figure 1:**
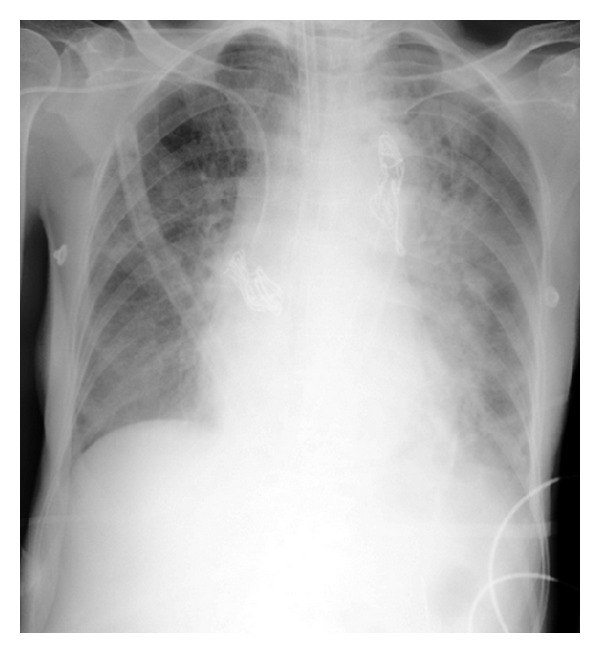
Chest X-ray showing a mass in the left upper lung field (Palla's sign).

The patient was a 64-year-old woman presented with progressive generalized weakness and dyspnea for 6 months. She had no risk factors for thromboembolism. She had history of headache, weight loss, and nonproductive cough prior to the admission and one episode of presyncope last year. Her vital signs on admission were a temperature of 38°C, blood pressure of 120/70 mmHg, pulse rate of 90 beats per minute and respiration rate of 24 breaths per minute with O_2_ saturation 99%, partial arterial blood gas pressures of 26 mmHg, and PCO2 of 40 mmHg. On physical examination, no clinical evidence of deep vein thrombosis was found and in cardiac auscultation, a systolic murmur (grade III/VI) was heard in lower left sternal border. Chest X-ray showed moderate enlargement of right atrium and dilatation of right descending pulmonary artery (Palla's sign) (Figure 1). CA 125, CA 99, and CEA were within normal range.

No abnormality was seen in her electrocardiography. Transthoracic echocardiography (ECG) showed a severe pulmonary artery hypertension (the predicted pulmonary artery pressure was 120 mmHg) with severe right ventricular hypertrophy, severe dysfunction, and moderate to severe tricuspid valve regurgitation. Main pulmonary artery (MPA) was occupied by a large nonhomogeneous mass with a very small flow from left side of MPA and significant stenosis (peak Gradient = 55 mmHg).

**Figure 2:**
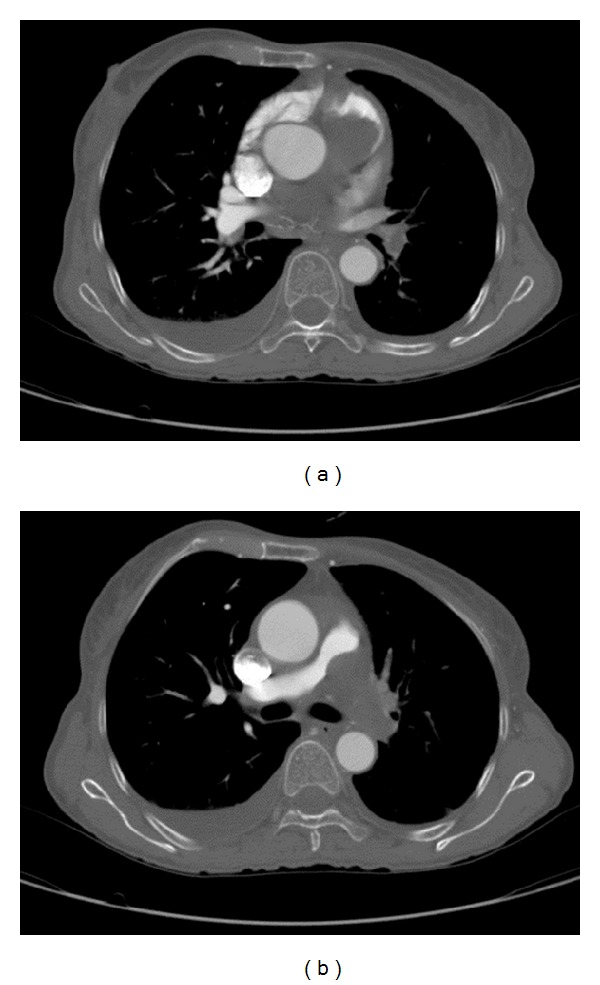
CT scan showing lobulated heterogeneous left hilar mass.

A contrast-enhanced computed tomography (CT) scanning of the chest showed lobulated heterogeneous left hilar mass occupying the precarinal and subcarinal space and invaded into left main pulmonary artery and pulmonary trunk. Right ventricle and right atrium strain and enlargement, mild pericardial effusion, and right sided pleural effusion were seen (Figure 1). The CT scan suggested malignant tumoral lesion like lymphoma, angiosarcoma, or bronchogenic carcinoma (Figure 2).

In thoracic MRI, both sided pleural effusion was seen. There was a polypoid lesion at trunk of the pulmonary with extension to left main and first-order expansion of lumen. No obvious extraluminal extension and significant enhancement were detected. Also, mild right ventricle and atrium dilatation were observed. The MRI findings suggested pulmonary thromboembolism, while tumoral lesion like angiosarcoma should be rolled out.

With the primary diagnosis of main pulmonary thromboembolism, the patient underwent pulmonary endarterectomy (PEA) through midsternotomy. On opening the pulmonary artery, a firm yellowish-shiny mass solid was found in pulmonary trunk. Gross examination showed creamy to gray color irregular tissue fragments with elasticus consistency and hemorrhagic change totally measured 7 × 6 × 1.5 cm. Complete gross tumor resection was performed and the mass was endarterectomized from the pulmonary artery and its branches. The aorta was cross-clamped for 86 minutes while the cardiopulmonary bypass time was 149 minutes. The patient received 2500 cc fluids during the operation and hematocrit was 23% at the time of removing the pump and was transferred to ICU after homeostasis. She was intubated for 26 hours and, 22 hours after the operation, she was extubated. 

**Figure 3:**
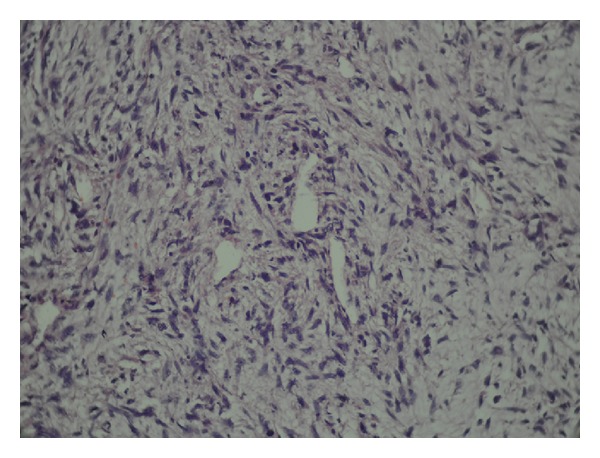
The intimate relationship of the tumor cells with the vessel walls is a clue to the diagnosis of leiomyosarcoma.

**Figure 4:**
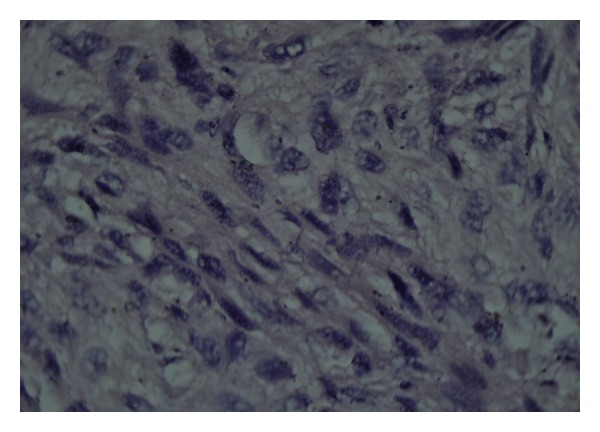
Cytologic features of leiomyosarcoma showing eosinophilic cytoplasm and blunt-ended nuclei.

**Figure 5:**
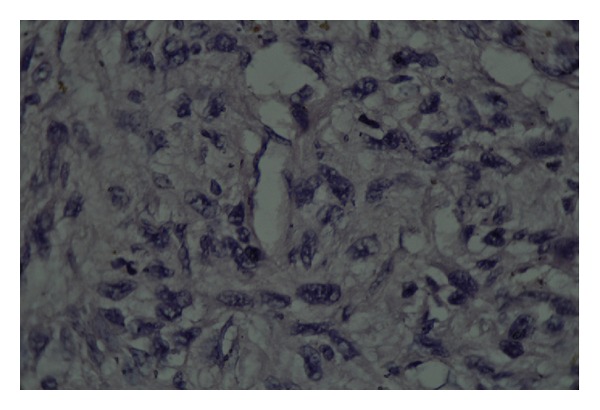
Cytologic features of leiomyosarcoma showing perinuclear vacuoles.

The pathology result confirmed primary pulmonary artery sarcoma with smooth muscle differentiation compatible with leiomyosarcoma (i.e., intimal sarcoma). Microscopically, the tumor was composed of spindle-shaped cell with pleomorphism and oval hyperchromatic to spindle nuclei was found. A pattern of tumor growth was observed in the lung parenchyma: tumoral cells arranged in parallel with whirling appearance forming storiform pattern. Mitotic activity was evaluated as the number of mitotic figures per 10 high-power fields was about 5-6 and bizarre cell and tumoral giant cells were present. Many cleft-like vascular channels in addition to hemorrhagic myxoid and necrotic changes were present. Pathological diagnosis confirmed leiomyosarcoma limited to pulmonary (Figures 3, 4, and 5).

Immunohistochemical staining was positive for smooth muscle actin (SMA), a marker for mesenchymal neoplasms. Immunohistochemical staining for factor 8, HMB 45, and CK was negative while it was focally positive for CD68 and 5100. Tumor differentiation score was 2.3 with mitosis count of 13 per 10 HPF and score of 2.3. D-dimer was 0.2 mg/dL (nl < 0.3 mg/dL). Tumor necrosis: less than 50% score: 1.2 and total score: 5.8. Grade: II.

The patient's postoperative course was uncomplicated, and she was discharged 19 days after operation. She was advised to perform daily respiratory physiotherapy in addition to abdominal and pelvic sonography and abdominal and pelvic CT scan. After discharge, the patient had respiratory problem and could not tolerate chemotherapy. So, 3 months after the operation, the patient was stable and chemotherapy with Adriamycin was prescribed but after one unsuccessful chemotherapy session, she was pronounced dead because of severe respiratory complications.

## 3. Discussion

Primary leiomyosarcoma cases of the pulmonary artery are extremely rare and most of them are initially misdiagnosed as pulmonary thromboembolism with symptoms of dyspnea, chest pain, cough, and hemoptysis [6, 7]. Both diseases are typically detected between the ages of 40 and 60, and women are involved twice as often as men. Physical examination, ECG, and the chest X-ray may not reveal abnormal findings. However, cardiomegaly and radiological signs of a peripheral hypoperfusion can be present if a large tumor mass is obstructing a main pulmonary artery vessel [8].

Atypical features such as lack of predisposing factors for thromboembolism, persistence of symptoms or recurrence despite adequate anticoagulation, and unilateral distribution of a massive perfusion defect may evoke the diagnosis of tumoral obstruction [9].

The differential diagnoses include pulmonary artery sarcoma, thromboembolism, and lung cancer. The symptoms of the pulmonary embolism are nonspecific and laboratory findings have a low diagnostic specificity and chest radiograph is generally nondiagnostic [4]. 

Our patient died 3 months after the operation. The median survival time of the pulmonary artery leiomyosarcoma patients has been reported to be 1.5 months. It is believed that early and primary surgical resection is the best treatment of choice and can prolong the patient's life to 10–12 months [7, 10]. 

The role of adjuvant therapy has not been yet clearly defined in the literature. The limited experience of any center in the treatment of these neoplasms makes it difficult to evaluate the relative importance of surgical excision and adjuvant therapy. Some investigators are in favor of adjuvant therapy and describe encouraging results [11, 12].

Scores derived from explicit prediction rules that combine clinical findings at presentation with predisposing factors have proved useful in determining the clinical or pretest probability of pulmonary embolism. Three scores (the Wells, Geneva, and revised Geneva score) have been recommended as diagnostic criteria [4, 13].

Our patient experienced only a slight dyspnea before admission, and her physical examination on admission produced no findings suggestive of pulmonary embolism. It is suggested that pulmonary artery sarcomas should be strongly suspected in cases who present with mass lesions in the pulmonary arteries but score low on the clinical prediction indexes. Several indicators on CT and MRI favor the diagnosis of pulmonary artery sarcoma over chronic thromboembolic disease [13]. Chest X-ray showed moderate enlargement of right atrium and dilatation of right descending pulmonary artery and MRI revealed a polypoid lesion at trunk of pulmonary artery with extension to left main pulmonary artery and its first branch. It would be better if we could perform PET scan to see the probable accumulation of 18F-fluorodeoxyglucose in the mass in pulmonary artery to confirm the presence or absence of malignant neoplasm, but unfortunately we do not have PET (Positron emission tomography) scan equipment in Qom and could not evaluate the patient by PET scan.

Differential diagnosis also included primary and metastatic lung cancer. Our patient never smoked and her serum tumor markers were all within normal ranges. It is said that the actual prevalence of pulmonary artery sarcoma is much higher than the estimated prevalence because of difficult diagnosis and often going unidentified without autopsy [4]. The prognosis for patients with pulmonary artery sarcoma is poor and in most cases, the mean survival time is less than 2 years. The reported case survived only for 3 months. Effect of chemotherapy or radiation therapy on prognosis is unclear and radical surgical resection seems to provide the only hope of long-term survival [14].

In this study, after the operation, patient was admitted at ICU for 19 days and she was intubated for 16 hours. Jamieson and colleagues in 2003, in evaluation of 1,500 pulmonary endarterectomy cases, found that the median stay in the intensive care unit was 96 hours, and the median number of days the patients were intubated was 1 ± 9.6 (longest, 86 days). The median length of hospital stay postoperatively was 10 days [15]. The prolonged length of hospital stay postoperatively for our patient may be due to packed sternum area's bleeding for which she was transferred to operation room for usual sternum closure.

Postoperative care is similar to that of routine open heart operations, except that aggressive diuresis is instituted to remove fluid in the third space that accumulated as a result of prolonged bypass and hypothermia and to reduce the incidence of pulmonary edema [16].

## 4. Conclusion

Initially, the reported patient underwent surgery due to pulmonary embolism but during the operation, the observed mass increased the probability of pulmonary artery neoplasm. Clinicians must consider pulmonary artery sarcoma when making the differential diagnosis for patients with pulmonary arteries masses. In addition to clinical prediction scores and CT and MRI findings to identify the patients with pulmonary artery sarcoma, PET scanning is the diagnosis of choice in differentiating embolism and neoplasm and is strongly recommended in these patients.
